# Supplementary material for: Dealing with being prescribed cardiovascular preventive medication: a narrative analysis of qualitative interviews with patients with recent acute coronary heart disease in Sweden
Source: BMJ Open. 2021 Dec 17;11(12):e056677. doi: 10.1136/bmjopen-2021-056677 (PMC8685966; doi:10.1136/bmjopen-2021-056677)
Supplement: Author's manuscript [file bmjopen-2021-056677.draft_revisions.pdf]

BMJ Open

BMJ Open is committed to open peer review. As part of this commitment we make the peer review history of every article we publish publicly available.

When an article is published we post the peer reviewers' comments and the authors' responses online. We also post the versions of the paper that were used during peer review. These are the versions that the peer review comments apply to.

The versions of the paper that follow are the versions that were submitted during the peer review process. They are not the versions of record or the final published versions. They should not be cited or distributed as the published version of this manuscript.

BMJ Open is an open access journal and the full, final, typeset and author-corrected version of record of the manuscript is available on our site with no access controls, subscription charges or pay-per-view fees (http://bmjopen.bmj.com).

If you have any questions on BMJ Open's open peer review process please email info.bmjopen@bmj.com

BMJ Open

Being prescribed cardiovascular preventive medication – a narrative analysis of interviews with patients with recent acute coronary heart disease.

Journal:	*BMJ Open*
Manuscript ID	bmjopen-2021-056677
Article Type:	Original research
Date Submitted by the Author:	13-Sep-2021
Complete List of Authors:	Hultberg, Josabeth; Linköping University; Region Östergötland, Åby Primary Health Care Centre<br>Nilsson, Staffan; Linkopings universitet Institutionen for medicin och halsa<br>Rudebeck, Carl Edvard; Kalmar County Council, Research Unit<br>Köhler, Anita Kärner; Linköping University
Keywords:	PRIMARY CARE, Coronary heart disease < CARDIOLOGY, PREVENTIVE MEDICINE, QUALITATIVE RESEARCH

SCHOLARONE™
Manuscripts

I, the Submitting Author has the right to grant and does grant on behalf of all authors of the Work (as defined in the below author licence), an exclusive licence and/or a non-exclusive licence for contributions from authors who are: i) UK Crown employees; ii) where BMJ has agreed a CC-BY licence shall apply, and/or iii) in accordance with the terms applicable for US Federal Government officers or employees acting as part of their official duties; on a worldwide, perpetual, irrevocable, royalty-free basis to BMJ Publishing Group Ltd ("BMJ") its licensees and where the relevant Journal is co-owned by BMJ to the co-owners of the Journal, to publish the Work in this journal and any other BMJ products and to exploit all rights, as set out in our licence.

The Submitting Author accepts and understands that any supply made under these terms is made by BMJ to the Submitting Author unless you are acting as an employee on behalf of your employer or a postgraduate student of an affiliated institution which is paying any applicable article publishing charge ("APC") for Open Access articles. Where the Submitting Author wishes to make the Work available on an Open Access basis (and intends to pay the relevant APC), the terms of reuse of such Open Access shall be governed by a Creative Commons licence – details of these licences and which Creative Commons licence will apply to this Work are set out in our licence referred to above.

Other than as permitted in any relevant BMJ Author's Self Archiving Policies, I confirm this Work has not been accepted for publication elsewhere, is not being considered for publication elsewhere and does not duplicate material already published. I confirm all authors consent to publication of this Work and authorise the granting of this licence.

For peer review only

Being prescribed cardiovascular preventive medication – a narrative analysis of interviews with patients with recent acute coronary heart disease.

Authors
Josabeth Hultberg[1,2], Staffan Nilsson[1], Carl Edvard Rudebeck[3], Anita Kärner Köhler[1]

[1]Department of Health, Medicine and Caring Sciences, Linköping University, Linköping, Sweden
[2]Åby Primary Health Care Centre, Norrköping, Sweden
[3]Research Unit, Kalmar County Region, Kalmar, Sweden

Corresponding author at:
E-mail: josabeth.hultberg@gmail.com

Telephone: +46 70 665 4275

Mail: Josabeth Hultberg, Primary Health Care Centre Åby, Box 75, SE-616 21 Åby, Sweden

Key words: Prescribing**,** Cardiovascular Diseases**,** Patient Perspectives

Word count: 3825

Abstract

Objective: To explore how patients with experience of acute coronary heart disease make sense of, and deal with, the fact of being prescribed cardiovascular preventive medication.

Design: Narrative interview study.

Setting: Swedish primary care.

Participants: Twenty-one participants with experience of being prescribed cardiovascular preventive medication, recruited from a randomized controlled study of problem-based learning for self-care for coronary heart disease.

Methods: The participants were interviewed individually 6-12 months after their hospitalization for acute coronary disease. A narrative analysis was conducted of their accounts of being prescribed cardiovascular preventive medication.

Results: Four themes shape the patients' experiences: "A matter of living" concerns an awareness of the will to live linked to being prescribed cardiovascular preventive

For peer review only - http://bmjopen.bmj.com/site/about/guidelines.xhtml

medication regarded in the light of the recent experience of acute coronary disease. In "Reconciliation of conflicting self-images", patients dealt with being prescribed preventive medication through work to restore an identity of someone responsible, in spite of viewing the taking of medication as questionable, and a transition from healthy to ill, in spite of medication being a symbol of disease. "Being in the hands of expertise" is about the seeking of an answer from a reliable prescriber to the question: 'Is this medication really necessary for me?' Existential labour was done to establish that the practice of taking cardiovascular preventive medication was an inevitable necessity, rather than an active choice. "Taking medicines no longer a big deal" could be the resulting experience of this process.

Conclusions: Adopting the practice to take cardiovascular preventive medication is facilitated by the resolution of existential challenges, the reconciliation of conflicting self-images, and the settling of the question of necessity of taking the medication in dialogue with a trusted prescriber.

Strengths and limitations of this study

- Through narrative analysis of interviews, this study explores the patient perspective of being prescribed cardiovascular preventive medication, and how it is dealt with in everyday life.
- A wide range of experiences of being prescribed cardiovascular preventive medication is represented in the data following the selection of participants from a study of self-care after hospital care for acute coronary heart disease.
- Swedish health care has a lower proportion of general practitioners than similar countries, and only a minority of the population is registered with a regular GP, which may limit the transferability of the findings.
- The interviews were conducted within a year of an episode of acute coronary heart disease, so their accounts may be coloured by psychological reactions to the relatively recent serious event.

INTRODUCTION

For peer review only

Cardiovascular preventive medication (CPM) is widely used, and prescribed mainly in primary care. Previous work has shown that patients tend to be reluctant to use medicines.[1,2] Patients with chronic conditions, including the risk of cardiovascular disease, manage their conditions and treatments every day. Consideration of the *burden of treatment*, the continuous and sometimes arduous work taken on by patients with chronic conditions, is increasing.[3,4] Based on studies of layman views on statins, a framework has been proposed in which the perceived necessity to use medication is weighed against potential concerns. [2,5] Patients who are prescribed long-term medication must navigate among paradoxical views on medication.[6] Seeing preventive medication as responsible and healthy behaviour stands in contrast to the view that taking medicine may be dangerous, and a morally dubious alternative to healthy habits.[7,8] Patients' arguments against taking CPM include that they rely on their body and its natural processes, and don't want to disturb it with medication.[9] Patients who use CPM in spite of their scepticism towards it argue that they are "pill-takers" out of necessity.[8] Being prescribed long-term medication, also for prevention, may lead to a changed self-perception that invokes a rewriting of one's biography.[10,11] Patients display resistance, not only to *taking medication*, but also to *becoming someone who does* .[8,10,12] The resistance to taking medicines may be intertwined with a resistance towards adopting an identity as a chronically ill person.[7]

Patients' reluctance to take medicines, and their experienced efforts while doing so, must be taken into account by prescribers. Studies of communication in actual consultations shed light on how patients execute their agency in decision-making through subtle resistance to treatment proposals, but also how they appreciate the support of prescribers to express their preferences.[12,13] The inherent uncertainty of CPM prescribing, where a calculated risk rather than a manifest symptomatic condition is the indication for treatment, is particularly challenging.[14,15] There is no unified definition of what shared decision-making (SDM) actually is, but its aim - to balance medical recommendations and the individual preferences of patients - makes great sense.[16] However, the assumption that a treatment decision is a distinct choice between neutrally formulated alternatives, occurring within the framework of a single consultation, has been contested.[17] Models that focus solely on the narrow context of individual consultations disregard the wider context of the everyday realities

For peer review only - http://bmjopen.bmj.com/site/about/guidelines.xhtml

experienced by patients. This may cause *contextual errors* in decision-making[18], which may increase the burden of treatment.[3] Common interpretations of SDM involve simplistic applications of autonomy ethics, and neglect the inherent asymmetry in patient-physician relationships.[19] It is not always realistic to expect patients to make independent rational decisions about medical treatments.[20] Decision aids, such as SCORE[21], which explicitly quantify risk and include inherent uncertainties, have been notoriously difficult to implement in clinical practice.[22] Despite this, current guidelines recommend that SDM is used, and it has even been suggested in policies for CPM that it should be mandatory.[21,23]

Physicians need guidance by relevant research to improve their skills in prescribing, which include skills in patient-doctor communication, and decision-making about treatments in the consultation.[24] Studies of prescribing in consultations are necessary but not sufficient.[12,13,17] Understanding how prescriptions of cardiovascular preventive medication play out in patients' lives will help physicians fulfil their role as prescribers.[3,18] This study aimed to explore how patients with experience of acute coronary heart disease make sense of, and deal with, the fact of being prescribed cardiovascular preventive medication.

Materials and methods

Patient and public involvement

No patient involved.

Setting and participants

Twenty-one participants were recruited consecutively from COR-PRIM, a primary care study of self-care after acute coronary heart disease.[26] Within the COR-PRIM study population, a wide variety of experiences of previous prescriptions of CPM is to be expected, making it a suitable sample for the purpose of our study. All participants had been admitted to hospital for acute coronary heart disease 6-12 months prior to the interview, and all gave written consent to participate after receiving information about the study.

Table 1. Participant characteristics, N = 21

Characteristics	n
**Sex**	
Male	17
Female	4

For peer review only - http://bmjopen.bmj.com/site/about/guidelines.xhtml

**Age, years**	72 (52-86)[1]
**Residential area**	
City	8
Rural or small town	13
**Education**	
Compulsory education[2]	8
Upper secondary school	6
University	7
**Marital status**	
Cohabitating	16
Living alone	5

[1] Median (min-max)
[2] Less than 10 years in school

For peer review only

The wives of six of the participants were present during the interviews, and supported their husbands to recall events and express their experiences.

JH interviewed the participants in their homes. They were asked to recall a situation in which cardiovascular preventive drugs had been discussed or prescribed. Drugs considered were antihypertensive medication, statins, and antiplatelet drugs such as acetylsalicylic acid. Follow-up questions were asked to explore relevant topics in greater depth. Figure 1 presents the interview guide used. Interviews lasted for 45-90 minutes, and were audio-recorded and transcribed verbatim by JH.

Analysis

We chose a narrative approach to analyse patients' accounts of being prescribed CPM.[25] The narrative analysis concerned both content (*what* was told), and structure (*how* it was told) as described by Reissman.[25] In the initial reading of the transcriptions, we looked for salient features of the material as a whole, with a narrative framework in mind. Structural elements (Figure 2) served to identify narratives.[27] The prescription of CPM was the complicating action around which these narratives revolved. The narratives described both the prescription of CPM after a recent cardiovascular event, and previous occasions on which CPM had been prescribed. Some interviews contained more than one narrative. A synopsis of each interview was made, in which the narratives were reproduced in condensed form. These condensed narratives were analysed in greater depth to define experiences, deliberations, and actions of the participants related to the fact that they were being prescribed CPM. Structural features such as the

For peer review only - http://bmjopen.bmj.com/site/about/guidelines.xhtml

framings and evaluations of the narrative material displayed their sense-making of reported speech.[27]

The narratives were rich in quotes. The participants told about what had been said, but also what may be said in a future scenario, by characters such as prescribers, other physicians, nurses, relatives and friends. They quoted texts, such as information leaflets. They also quoted themselves, both actual utterances, and their thoughts: their inner voices. Opinions and experiences of phenomena in the narratives became evident through internal evaluations.[27] Some, for example, used a mocking tone of voice when quoting someone they did not agree with. The analysis of the different voices used by the participants rested on Goffman's analytical framework for aspects of narrators, including the concept of *"footing".* This framework helps to reveal the speakers' positioning of themselves in relation to reported speech and events.[28]

Tentative themes within one interview were tried against each other, and then against those from other interviews. Similar tentative themes were brought together into more general themes, which were then further elaborated in an iterative process. In a final step, the themes were checked for their bearing on the original interview data, and adjusted to provide the final themes of the study. In each step, JH made an initial analysis. All authors discussed the emerging results. Throughout the process, the analytical focus was to capture the participants' ways of dealing with the fact that they were being prescribed CPM.

Results

Four themes became apparent during the analysis: "a matter of living", "reconciliation of conflicting self images", "being in the hands of expertise", and "taking medicines no longer a big deal". Quotes, including longer passages, were selected to illustrate the themes, and to display the dialogic and narrative structure of the data. The interviews were numbered 1-21, in the order they were conducted.

A matter of living

Implicit in the recommendation for preventive treatment is its importance for survival. Such a message may evoke an awareness of being mortal, and the fundamental will to live. Dealing with the awareness of mortality is not merely about avoiding death, but

For peer review only - http://bmjopen.bmj.com/site/about/guidelines.xhtml

involves reflections about what makes life worth living, and what it means to lead a meaningful life. Staying alive to continue relationships with loved ones was salient:

I didn't use to take that much medication but now I have to.

Interviewer: Who makes you?

The COPD (chronic obstructive pulmonary disease) and heart

Wife: Basically, he wants to live ...

... with you! (looks at wife) (#12)

The awareness of being mortal that was evoked by being prescribed CPM stimulated efforts to strike a balance between the pros and cons of taking medication. For example, side effects could be accepted with the argument that the medication was regarded lifesaving:

It is important that I take this and I don't care if it makes me feel a bit sick, I have to or else I will die. (#14)

Some participants held fundamental objections to taking CPM, but experienced even so gratitude for having the chance to stay alive, which was attributed to the medication:

... Now you're a bit scared and think: well, I'd better take it for the heart, that's what they said ... so of course you're glad for all these pills and that they work in the body. (#2)

Reconciliation of conflicting self-images

Most participants were generally averse to taking medicines, and held the view that they are generally overprescribed and overused. The participants typically presented themselves as sceptical towards medication, not prone to use it, and stated that medicines should only be taken if necessary. Being given a prescription and becoming a "pill-taker" challenged this self-image. Most participants had been prescribed CPM for primary or secondary prevention, prior to the recent hospitalisation for acute coronary heart disease. Both participants who had been prescribed medication for primary prevention before having had symptomatic cardiovascular disease, and those who didn't have it until after such disease, became aware that they were at risk of future cardiovascular disease. They perceived that their status changed from healthy to being in need of correction through medical treatment:

For peer review only - http://bmjopen.bmj.com/site/about/guidelines.xhtml

(Now) I have to. I take it for self-preservation, but like I say: I don't like to put chemicals into my body. (#3)

One way of dealing with the new conditions was to seek to preserve the "only-when-necessary" view of self. Taking CPM was framed as an inevitable consequence of the state of health, not as an active choice. A face-saving rhetoric indicated that this was a sensitive topic, which evoked feelings of embarrassment and shame. Potential blame for having caused the state of being at risk was countered by a defensive line of argument. Participants emphasised that they had been struck by disease, or had high cholesterol values, in spite of a healthy lifestyle. Having smoked was mentioned, but downplayed: *"I used to smoke but I quit … I used to drink a lot of milk … I think that was the biggest cause" (#21).* Hard work, and stressful life events were common explanations. Heredity was another: *"These bloody sickness-genes" (#11).* One line of defence was to claim to be an exception in comparisons with others: *"Many get way too much medicine, but that's perhaps more in nursing homes" (#4),* while another was to normalise the condition: "*Now it (angioplasty) is common, they go: 'Oh you did it too?' … In the nineties you went down in the basement of the University Hospital. It was like coming to Hades in the underworld and was serious as hell. They did a couple a week, now they do several a day." (#7)*

Being in the hands of expertise

A personal relationship with a prescriber was essential for patients in their work to seek reassurance that CPM was necessary. Some had experienced a continuous relationship with the physician responsible for prescribing their medication, but this was not the case for most. Without such a relationship, patients experienced feelings of abandonment: "*They took away furosemide and that other one, and I lacked … they just said: 'You can see how you feel', but how and when, with whom should I talk about how I feel? I didn't have anyone to talk with; it was all up to me." (#15)*

Being in the hands of expertise and *feel* powerless may not *be* powerless, when patients take a position in which they actively accept being cared for. Patients may choose to place the responsibility for decision-making on the prescribing doctor if they have a trustful relationship with the doctor, and in this way resolve their doubts about whether

For peer review only - http://bmjopen.bmj.com/site/about/guidelines.xhtml

it is necessary to take medication: "*You look up to someone who knows everything about this, and you are grateful that someone sort of weighs and measures you, and corrects and changes the medicines. It's like going to the dentist. You're helpless. What if the dentist says: 'Hey this tooth has to come out'. You don't ask: 'Do I really have to?'. Or at the garage, you don't have a clue; the car mechanic can say anything. So it feels a bit like you are powerless." (#1)*

The question *"Do I really have to take it?"* occurred frequently in the narratives. Reassurance was sought in dialogue with a prescribing physician, who could explain sufficiently clearly why the medication was recommended, and who was willing to take the responsibility to state that it was necessary. The attitude towards the authority of expertise ranged from more-or-less considered acceptance and trust: "*You have to trust what they say, and they know that a lot better than I do anyway." (#4)*, to critical scepticism: "*It is in my nature. I don't just accept anything" (#16).* Many narratives illustrated how important it was to have an expert to turn to, regardless of how the patient viewed the authority of expertise. This was particularly the case when such an expert was lacking. Some patients were aware of treatment guidelines, and that the expert community has different views on treatment indications. In spite of this, and in spite of an expressed request for information, patients described themselves to be in the hands of the medical expertise. Most were content with simply the possibility of dialogue: "*You don't pick a fight about it and say: 'I won't take this', or: 'I want this that I read about'. No, you take it for granted that this is a competent person." (#19)*

Taking medicines no longer a big deal

The concluding remarks in many narratives showed that an attitude in which taking medicines was no longer a big deal had been acquired. Medication had been incorporated into ordinary life:

They said you had to take this ... for everything to work and for the blood clots that can build up and all that, and I have brochures about it that I can read if I want, but I think now when you have them sitting there (nods towards the medicine packages on the kitchen shelf) it goes automatically, every night and every morning. (#2)

For peer review only - http://bmjopen.bmj.com/site/about/guidelines.xhtml

For some patients, a no-big-deal attitude came with minimal effort or reflection, whereas most had put considerable work into dealing with being prescribed CPM. This process of becoming a "pill-taker" without it being a big deal seemed to be facilitated by the resolution of existential challenges, the reconciliation of conflicting self-images, and the conclusion, after dialogue with a trusted prescriber, that it was necessary. The recent coronary heart event further lessened any hesitation about taking CPM, and motivated the view that it was truly necessary.

Nobody had a clue before this happened or talked about me belonging to a risk group, but now after getting the stent, I obviously do, so that is something to think about (when deciding about cholesterol treatment). (#19)

Some patients who were still in the process of dealing with one or several aspects of being prescribed CPM experienced their medication as a reminder of their vulnerability. It was difficult to ignore these feelings. Taking the medicines was still burdensome, and not yet an effortless daily routine:

(Shows that she has put the medicine dispenser out of sight in a cupboard) Sometimes when it is time for medication, and there are people over, I think I mustn't forget to take it when they have left, because I would never … Yak, it's so dreadful … Perhaps it takes a year to grasp, before everything has calmed down, all of this, and you make it sort of into a ritual after a while. (#1)

Discussion

Dealing with being prescribed CPM involved the themes: "a matter of living", "reconciliation of conflicting self-images", "being in the hands of expertise", and "taking medicines no longer a big deal". Patients worked to orient towards living rather than survival, and to find a coherent view of self as someone who must take CPM. They sought an answer from a reliable prescriber to the question: Is this medication really necessary for me? This implies that patients may execute their autonomy by actively placing the responsibility for the decision about CPM with the prescriber. A successful process established that taking CPM was necessary. The process by which taking medicines was established as necessary seemed to facilitate the incorporation of it into

For peer review only - http://bmjopen.bmj.com/site/about/guidelines.xhtml

everyday life. A stalled process of making it necessary appeared as a particular burden of treatment.

The wide range of experiences that the participants displayed is a strength of this study. Further, previous prescriptions were contrasted to the most recent ones, and reflected in the light of the recent hospitalisation, which contributes to the credibility of the results. The interviews were conducted within a year of an episode of acute coronary heart disease, and the participants may have still been experiencing reactions to the serious event, which may have resulted in their focus on existential matters.[29] We did not differentiate between experiences of primary and secondary prevention, in line with previous studies of patient perspectives when deciding whether to take CPM.[5,8] The participants in COR-PRIM rated high on scales that measure patient empowerment and general self-efficacy.[30] They may be inclined to share their viewpoints to improve healthcare, and thus be particularly well-suited to give voice to experiences of receiving prescriptions for CPM. All four of the themes appeared in almost every interview, which suggests that the themes have a general relevance. The presence of partners during some of the interviews was not planned, but turned out to be advantageous. As noted in a similar study, the dialogues of the couples added perspectives to the accounts, while keeping a respectful focus on the experiences of the patient.[8] Some participants showed symptoms of disease: in some cases this included signs of cerebrovascular disease with some cognitive impact. This is to be expected in this group of patients. It is important, and a strength of the study that the voices of also these patients are heard, something that this study has made possible. The presence of partners facilitated this. We believe that some of those who chose to participate in the research projects did so as a consequence of the lack of a personal relationship with a prescribing physician that many participants described. Health care in Sweden has a lower proportion of general practitioners than similar countries, and only a minority of the population are registered with a regular GP.[31] This may reduce the transferability of the findings. On the other hand, the negative experiences of lacking a continuous personal relationship with a prescriber that was clearly voiced by the participants suggest that this is of general importance.

For peer review only - http://bmjopen.bmj.com/site/about/guidelines.xhtml

The general reluctance towards medication was overridden by the will to live, with an understanding of taking CPM as necessary to survive. It was framed as an inevitable must, not as a choice. Polak *et al.* described this standpoint as a defence of morality, i.e. against the accusations of leading an unhealthy life style. This agrees with the defensive rhetoric that many of the participants took, and the way in which they dealt with the need for CPM as a morally sensitive issue.[8] The framing of it being necessary to take CPM appeared also to overrule uncertainty, making it into a question of life or death. The effects of CPM are overestimated by both laymen and general practitioners, which may be a result of this concept of necessity.[5,15] The notion of having no choice, following the necessity framing, is inconsistent with shared decision-making models that are all about choosing between alternatives. Those who are prescribed CPM must deal with a challenge to the self-image, and self-perception changes when long-term medication is prescribed.[10] Some of the resistance to taking medication may stem from a denial of being ill.[1] However, also medication prescribed for prevention, in the absence of symptomatic disease, may call for a rewriting of the biography.[11] The key to the reconciliation of the conflicting images held by the patients in our study was the work with identity by which they established that it was necessary to take CPM, and that they had become a medicine-taker.

Our findings indicate that patients want information and explanations from the prescriber about why CPM has been prescribed, but want the prescriber to determine whether it is necessary. Previous work has shown that patients prefer to take part in the decision-making process, but do not to make the final decision.[32,33] Patients can in this way execute their agency and autonomy, by asking for information and a dialogue but deferring the responsibility for the decision.[12] Prescribers must be aware of the inherent asymmetry in a patient-doctor relationship, and must be receptive to the patients' active deferral of responsibility for decision-making. They must strike a balance: to avoid violating patient autonomy without abandoning them.[34,35] Patient autonomy in this situation is not absolute, but depends on the response of the prescriber.[36,19] Elderly patients who take several medicines are more likely to find the medication necessary if they interact with a "good" doctor.[32] Even healthcare professionals, when patients, depend on the relationship with doctors and nurses, find rest in their hands, and trust

For peer review only - http://bmjopen.bmj.com/site/about/guidelines.xhtml

that they know what they're doing.[37] Plain information from a faceless expert community causes even better-informed patients to feel abandoned.[38] The process of making the taking of CPM to be necessary may result in the taking being incorporated into ordinary everyday life, even though it may threaten the "ordinariness" for persons who view themselves as non-pill-takers, and those who view medicines as a necessary evil.[7,8]

The implicit or explicit question from a patient: "Is it really necessary for me to take CPM?" requires a definitive answer. The submission to expertise may be a conscious and autonomous act by which responsibility for decisions is placed on the professional. If the prescriber shows no interest in the patient's expectations in this regard, the patient may feel abandoned. A personal continuous relationship with the prescriber helps patients to express their needs during the process that leads to a decision, and eventually to a no-big-deal attitude towards taking CPM. As Julian Tudor Hart put it: *"Effective health care has to be built around real continuing personal stories, not episodic fragments of standardized process."*.[39] In the same spirit, the concept of shared understanding has been proposed, in an attempt to merge SDM, evidence-based medicine, and patient-centred medicine. Attentive listening to patients' narratives is essential to the practice of shared understanding.[24,40]

Conclusions

Patients' unmet existential needs when being prescribed CPM add to the burden of treatment. A continuous and trustful relationship with the prescribing doctor may facilitate the reconciliation of conflicting self-images, and the settling of the question of whether the medication is necessary.

Conflicts of interests statement

The authors declare no conflicts of interests.

Ethical approval was obtained from the Regional Ethics Committee of Linköping, Ref. No. 2014/5-31.

For peer review only - http://bmjopen.bmj.com/site/about/guidelines.xhtml

Author contributions

AKK and SN were responsible for the recruitment of participants. All authors contributed to the design of the study. JH performed and transcribed the interviews. All authors contributed to the analysis of the results. JH wrote a manuscript draft, which all authors critically revised.

Funding

This study was supported by the County Council of Östergötland (no grant number) and Lions forskningsfond mot folksjukdomar (Lions Sweden, Districts 101-V, 101-S, 101-O and Linköping University Medical Faculty, no grant number).

Data availability

No additional data available.

Acknowledgements

We are indebted to the participants for their generosity with their time and stories, and for their hospitality during the interviews.

For peer review only

Tables and figures

Table 1 Participant characteristics.

Figure 1 Interview guide.

Figure 2 Structural elements in narratives according to Labov & Waletsky.

REFERENCES

1 Pound P, Britten N, Morgan M, *et al.* Resisting medicines: a synthesis of qualitative studies of medicine taking. *Soc Sci Med 1982* 2005;**61**:133–55. doi:10.1016/j.socscimed.2004.11.063

2 Horne R, Weinman J, Hankins M. The beliefs about medicines questionnaire: The development and evaluation of a new method for assessing the cognitive representation of medication. *Psychol Health* 1999;**14**:1–24.

3 May CR, Eton DT, Boehmer K, *et al.* Rethinking the patient: using Burden of Treatment Theory to understand the changing dynamics of illness. *BMC Health Serv Res* 2014;**14**:281. doi:10.1186/1472-6963-14-281

4 Leppin AL, Montori VM, Gionfriddo MR. Minimally Disruptive Medicine: A Pragmatically Comprehensive Model for Delivering Care to Patients with Multiple Chronic Conditions. *Healthcare* 2015;**3**:50–63. doi:10.3390/healthcare3010050

5 Lytsy P, Burell G, Westerling R. Views on treatment necessity, harm, and benefits in patients using statins. *Med Decis Mak Int J Soc Med Decis Mak* 2010;**30**:594–609. doi:10.1177/0272989X09353196

6 Gillett J, Ross A. Confronting Medicine's Dichotomies: Older Adults' Use of Interpretative Repertoires in Negotiating the Paradoxes of Polypharmacy and Deprescribing. *Qual Health Res* 2019;:1049732319868981. doi:10.1177/1049732319868981

7 Eborall HC, Will CM. 'Prevention is better than cure, but ...': Preventive medication as a risk to ordinariness? *Health Risk Soc* 2011;**13**:653–68. doi:10.1080/13698575.2011.624177

8 Polak L. What is wrong with 'being a pill-taker'? The special case of statins. *Sociol Health Illn* 2017;**39**:599–613. doi:10.1111/1467-9566.12509

9 Köhler AK, Nilsson S, Jaarsma T, *et al.* Health beliefs about lifestyle habits differ between patients and spouses 1 year after a cardiac event - a qualitative analysis based on the Health Belief Model. *Scand J Caring Sci* 2017;**31**:332–41. doi:10.1111/scs.12351

10 Andén A, Andersson S-O, Rudebeck C-E. Satisfaction is not all – patients' perceptions of outcome of general practice consultations, a qualitative study. *BMC Fam Pract* 2005;**6**:43. doi:10.1186/1471-2296-6-43

11 Sångren H, Reventlow S, Hetlevik I. Role of biographical experience and bodily sensations in patients' adaptation to hypertension. *Patient Educ Couns* 2009;**74**:236–43. doi:10.1016/j.pec.2008.08.007

12 Hultberg J, Rudebeck CE. Patient participation in decision-making about cardiovascular preventive drugs – resistance as agency. *Scand J Prim Health Care* Published Online First: 28 February 2017.http://www.tandfonline.com/doi/full/10.1080/02813432.2017.1288814 (accessed 1 Mar 2017).

13 Landmark AMD, Gulbrandsen P, Svennevig J. Whose decision? Negotiating epistemic and deontic rights in medical treatment decisions. *J Pragmat* 2015;**78**:54–69. doi:10.1016/j.pragma.2014.11.007

14 Hultberg J, Rudebeck CE. Clinical Gaze in Risk-Factor Haze: Swedish GPs' Perceptions of Prescribing Cardiovascular Preventive Drugs. *Int J Fam Med*;**2012**:612572. doi:10.1155/2012/612572

15 Treadwell JS, Wong G, Milburn-Curtis C, *et al.* GPs' understanding of the benefits and harms of treatments for long-term conditions: an online survey. *BJGP Open* Published Online First: 4 March 2020. doi:10.3399/bjgpopen20X101016

For peer review only - http://bmjopen.bmj.com/site/about/guidelines.xhtml

16 Bomhof-Roordink H, Gärtner FR, Stiggelbout AM, *et al.* Key components of shared decision making models: a systematic review. *BMJ Open* 2019;**9**:e031763. doi:10.1136/bmjopen-2019-031763

17 Ofstad EH, Frich JC, Schei E, *et al.* Temporal characteristics of decisions in hospital encounters: A threshold for shared decision making? A qualitative study. *Patient Educ Couns* 2014;**97**:216–22. doi:10.1016/j.pec.2014.08.005

18 Weiner SJ, Schwartz A. Contextual Errors in Medical Decision Making: Overlooked and Understudied. *Acad Med J Assoc Am Med Coll* 2016;**91**:657–62. doi:10.1097/ACM.0000000000001017

19 Herlitz A, Munthe C, Törner M, *et al.* The Counseling, Self-Care, Adherence Approach to Person-Centered Care and Shared Decision Making: Moral Psychology, Executive Autonomy, and Ethics in Multi-Dimensional Care Decisions. *Health Commun* 2016;**31**:964–73. doi:10.1080/10410236.2015.1025332

20 Beauchamp TL, Childress JF. *Principles of biomedical ethics*. Oxford: : Oxford University Press 2001.

21 Piepoli MF, Hoes AW, Agewall S, *et al.* 2016 European Guidelines on cardiovascular disease prevention in clinical practice. *Atherosclerosis* 2016;**252**:207–74. doi:10.1016/j.atherosclerosis.2016.05.037

22 Légaré F, Adekpedjou R, Stacey D, *et al.* Interventions for increasing the use of shared decision making by healthcare professionals. *Cochrane Database Syst Rev* 2018;**7**:CD006732. doi:10.1002/14651858.CD006732.pub4

23 Merchant FM, Dickert NW, Howard DH. Mandatory Shared Decision Making by the Centers for Medicare & Medicaid Services for Cardiovascular Procedures and Other Tests. *JAMA* 2018;**320**:641–2. doi:10.1001/jama.2018.6617

24 Locock L, Lehman R, Epstein RM. Sharing Experiences of Illness and Care. *JAMA Intern Med* 2017;**177**:1249–50. doi:10.1001/jamainternmed.2017.1935

25 Riessman CK. *Narrative methods for the human sciences*. Los Angeles, Calif.: : Sage 2008.

26 Karner A, Nilsson S, Jaarsma T, *et al.* The effect of problem-based learning in patient education after an event of CORONARY heart disease--a randomised study in PRIMARY health care: design and methodology of the COR-PRIM study. *BMC Fam Pr*;**13**:110. doi:10.1186/1471-2296-13-110

27 Labov W, Waletzky J. Narrative analysis: Oral versions of personal experience. *J Narrat Life Hist* 1997;**7**:3–38. doi:10.1075/jnlh.7.02nar

28 Goffman E. *Forms of talk*. Philadelphia: : Univ. of Pennsylvania Press 2008.

29 Odell A, Grip L, Hallberg LR-M. Restenosis after Percutaneous Coronary Intervention (PCI): Experiences from the Patients' Perspective. *Eur J Cardiovasc Nurs* 2006;**5**:150–7. doi:10.1016/j.ejcnurse.2005.10.004

For peer review only

30 Kärner Köhler A, Tingström P, Jaarsma T, *et al.* Patient empowerment and general self-efficacy in patients with coronary heart disease: a cross-sectional study. *BMC Fam Pract* 2018;**19**:76. doi:10.1186/s12875-018-0749-y

31 International Health Policy Survey of Primary Care Physicians 2015. https://www.vardanalys.se/in-english/reports/international-health-policy-survey-of-primary-care-physicians-2015 (accessed 8 Aug 2020).

32 Moen J, Bohm A, Tillenius T, *et al.* "I don't know how many of these [medicines] are necessary.."—A focus group study among elderly users of multiple medicines. *Patient Educ Couns* 2009;**74**:135–41. doi:10.1016/j.pec.2008.08.019

33 Edwards A, Elwyn G. Inside the black box of shared decision making: distinguishing between the process of involvement and who makes the decision. *Health Expect* 2006;**9**:307–20. doi:10.1111/j.1369-7625.2006.00401.x

34 Morberg Jämterud S. Human Dignity : A Study in Medical Ethics. Published Online First: 2016.http://urn.kb.se/resolve?urn=urn:nbn:se:uu:diva-300409 (accessed 26 Nov 2018).

35 Lindström A, Weatherall A. Orientations to epistemics and deontics in treatment discussions. *J Pragmat* 2015;**78**:39–53. doi:10.1016/j.pragma.2015.01.005

36 Gulbrandsen P, Clayman ML, Beach MC, *et al.* Shared decision-making as an existential journey: Aiming for restored autonomous capacity. *Patient Educ Couns* Published Online First: 6 July 2016. doi:10.1016/j.pec.2016.07.014

37 Olthuis G, Leget C, Grypdonck M. Why shared decision making is not good enough: lessons from patients. *J Med Ethics* 2014;**40**:493–5. doi:10.1136/medethics-2012-101215

38 Johansson M, Jørgensen KJ, Getz L, *et al.* "Informed choice" in a time of too much medicine—no panacea for ethical difficulties. *BMJ* 2016;**353**:i2230. doi:10.1136/bmj.i2230

39 Hart JT. *The political economy of healthcare: where the NHS came from and where it could lead*. Bristol: : Policy Press 2010.

40 Maskrey N, Gordon A. Shared Understanding With Patients. *JAMA Intern Med* 2017;**177**:1247–8. doi:10.1001/jamainternmed.2017.1932

For peer review only - http://bmjopen.bmj.com/site/about/guidelines.xhtml

Figure 1. Interview guide

Topics to cover in interviews:

- Cardiovascular risk, risk factors, the concept of risk and the participant's own risk of cardiovascular disease
- Power, responsibility and participation in prescribing decisions
- Knowledge relevant for treatment decisions
- Communication between patients and healthcare professionals about medication

For peer review only

Figure 2. Structural elements in narratives according to Labov & Waletsky

**Abstract**	introduction of story
**Orientation**	background information, setting and participants in story
**Complicating action**	account for events in temporally ordered narrative clauses
**Evaluation**	revelations of attitudes towards the content of the story, what the point of the story is according to the narrator, why it makes sense, how it creates meaning
**Resolution**	the end result of the events
**Coda**	concluding remark

For peer review only

Dealing with being prescribed cardiovascular preventive medication: a narrative analysis of qualitative interviews with patients with recent acute coronary heart disease in Sweden.

Journal:	*BMJ Open*
Manuscript ID	bmjopen-2021-056677.R1
Article Type:	Original research
Date Submitted by the Author:	24-Nov-2021
Complete List of Authors:	Hultberg, Josabeth; Linköping University; Region Östergötland, Åby Primary Health Care Centre<br>Nilsson, Staffan; Linkopings universitet Institutionen for medicin och halsa<br>Rudebeck, Carl Edvard; Kalmar County Council, Research Unit<br>Köhler, Anita Kärner; Linköping University
**Primary Subject Heading**:	General practice / Family practice
Secondary Subject Heading:	Cardiovascular medicine, Communication, General practice / Family practice, Patient-centred medicine, Pharmacology and therapeutics
Keywords:	PRIMARY CARE, Coronary heart disease < CARDIOLOGY, PREVENTIVE MEDICINE, QUALITATIVE RESEARCH

I, the Submitting Author has the right to grant and does grant on behalf of all authors of the Work (as defined in the below author licence), an exclusive licence and/or a non-exclusive licence for contributions from authors who are: i) UK Crown employees; ii) where BMJ has agreed a CC-BY licence shall apply, and/or iii) in accordance with the terms applicable for US Federal Government officers or employees acting as part of their official duties; on a worldwide, perpetual, irrevocable, royalty-free basis to BMJ Publishing Group Ltd ("BMJ") its licensees and where the relevant Journal is co-owned by BMJ to the co-owners of the Journal, to publish the Work in this journal and any other BMJ products and to exploit all rights, as set out in our licence.

The Submitting Author accepts and understands that any supply made under these terms is made by BMJ to the Submitting Author unless you are acting as an employee on behalf of your employer or a postgraduate student of an affiliated institution which is paying any applicable article publishing charge ("APC") for Open Access articles. Where the Submitting Author wishes to make the Work available on an Open Access basis (and intends to pay the relevant APC), the terms of reuse of such Open Access shall be governed by a Creative Commons licence – details of these licences and which Creative Commons licence will apply to this Work are set out in our licence referred to above.

Other than as permitted in any relevant BMJ Author's Self Archiving Policies, I confirm this Work has not been accepted for publication elsewhere, is not being considered for publication elsewhere and does not duplicate material already published. I confirm all authors consent to publication of this Work and authorise the granting of this licence.

For peer review only

Dealing with being prescribed cardiovascular preventive medication: a narrative analysis of qualitative interviews with patients with recent acute coronary heart disease in Sweden.

Authors
Josabeth Hultberg[1,2], Staffan Nilsson[1], Carl Edvard Rudebeck[3], Anita Kärner Köhler[1]

[1]Department of Health, Medicine and Caring Sciences, Linköping University, Linköping, Sweden
[2]Åby Primary Health Care Centre, Norrköping, Sweden
[3]Research Unit, Kalmar County Region, Kalmar, Sweden

Corresponding author at:
E-mail: josabeth.hultberg@gmail.com

Telephone: +46 70 665 4275

Mail: Josabeth Hultberg, Primary Health Care Centre Åby, Box 75, SE-616 21 Åby, Sweden

Key words: Primary Care, Coronary Heart Disease, Preventive medicine, Qualitative Research

Word count: 3980

For peer review only

Abstract

Objective: To explore how patients with experience of acute coronary heart disease make sense of, and deal with, the fact of being prescribed cardiovascular preventive medication.

Design: Qualitative interview study.

Setting: Swedish primary care.

Participants: Twenty-one participants with experience of being prescribed cardiovascular preventive medication, recruited from a randomized controlled study of problem-based learning for self-care for coronary heart disease.

Methods: The participants were interviewed individually 6-12 months after their hospitalization for acute coronary disease. A narrative analysis was conducted of their accounts of being prescribed cardiovascular preventive medication.

Results: Four themes shape the patients' experiences: "*A matter of living*" concerns an awareness of the will to live linked to being prescribed cardiovascular preventive medication regarded in the light of the recent hospitalization. In "*Reconciliation of conflicting self-images*", patients dealt with being prescribed preventive medication through work to restore an identity of someone responsible in spite of viewing the taking of medication as questionable. The status of feeling healthy, while being someone in need of medication, also constituted conflicting self-images. Following this, taking medication was framed as necessary, not as an active choice. "*Being in the hands of expertise*" is about the seeking of an answer from a reliable prescriber to the question: 'Is this medication really necessary for me?' Existential labour was done to establish that the practice of taking cardiovascular preventive medication was an inevitable necessity, rather than an active choice. "*Taking medicines no longer a big deal*" could be the resulting experience of this process.

Conclusions:

For peer review only

Unmet existential needs when being prescribed cardiovascular preventive medication seem to be a component of the burden of treatment. A continuous and trustful relationship with the prescribing doctor may facilitate the reconciliation of conflicting self-images, and support patients in their efforts to incorporate their medicines taking into daily life.

Strengths and limitations of this study

- Through narrative analysis of interviews, this study explores the patient perspective of being prescribed cardiovascular preventive medication, and how it is dealt with in everyday life.
- A wide range of experiences of being prescribed cardiovascular preventive medication is represented in the data following the selection of participants from a study of self-care after hospital care for acute coronary heart disease.
- Swedish health care has a lower proportion of general practitioners than similar countries, and only a minority of the population is registered with a regular GP, which may limit the transferability of the findings.
- The interviews were conducted within a year of an episode of acute coronary heart disease, so their accounts may be coloured by psychological reactions to the relatively recent serious event.

INTRODUCTION

Cardiovascular preventive medication (CPM) is widely used and prescribed mainly in primary care. Patients tend to be reluctant to use medicines.[1,2] Patients with long-term medication manage their treatments every day. There is increasing consideration of the *burden of treatment*, the continuous and sometimes arduous work taken on by patients with long-term medication.[3,4] How patients formulate the pros and cons of CPM seems similar for primary and secondary prevention.[5] A framework has been proposed in which the perceived necessity to use medication is weighed against potential concerns.[2,6] Patients who are prescribed long-term medication navigate among paradoxical views on medication.[7] Seeing preventive medication as responsible and healthy behaviour stands in contrast to the view that taking medicine may be dangerous, and a morally dubious alternative to healthy habits.[8,9] Patients' arguments against taking

For peer review only - http://bmjopen.bmj.com/site/about/guidelines.xhtml

CPM include that they rely on their body's natural processes, and don't want to disturb it with medication.[10] Patients who use CPM in spite of their scepticism towards it argue that they are "pill-takers" out of necessity.[9] Being prescribed long-term medication, also for prevention, may lead to a changed self-perception that invokes a rewriting of one's biography.[11,12] Patients display resistance, not only to *taking medication*, but also to *becoming someone who does* .[9,11,13] The resistance to taking medicines may be intertwined with a resistance towards adopting an identity as a chronically ill person.[8]

Patients' reluctance to take medicines, and their experienced efforts while doing so, must be taken into account by prescribers. Studies of communication in actual consultations shed light on how patients execute their agency in decision-making through subtle resistance to treatment proposals, but also how they appreciate the support of prescribers to express their preferences.[13,14] The inherent uncertainty of CPM prescribing is particularly challenging, as the treatment aims to reduce the abstract risk of future disease, not to relieve symptoms.[15,16] There is no unified definition of what shared decision-making (SDM) actually is, but its aim - to balance medical recommendations and the individual preferences of patients - makes great sense.[17] However, the assumption that a treatment decision is a distinct choice between neutrally formulated alternatives, occurring within the framework of a single consultation, has been contested.[18] Models that focus solely on the narrow context of individual consultations disregard the wider context of the everyday realities experienced by patients. This may cause *contextual errors* in decision-making[19], which may increase the burden of treatment.[3] Common interpretations of SDM involve simplistic applications of autonomy ethics, and neglect the inherent asymmetry in patient-physician relationships.[20] It is not always realistic to expect patients to make independent rational decisions about medical treatments.[21] Decision aids, such as SCORE[22], which explicitly quantify risk and include inherent uncertainties, have been notoriously difficult to implement in clinical practice.[23] Despite this, current guidelines recommend that SDM is used, and it has even been suggested that it should be mandatory.[22,24]

Physicians need guidance by relevant research to improve their skills in prescribing, which include patient-doctor communication, and decision-making about treatments in

For peer review only - http://bmjopen.bmj.com/site/about/guidelines.xhtml

the consultation.[25] Studies of prescribing in consultations are necessary but not sufficient.[13,14,18] Understanding how prescriptions of cardiovascular preventive medication play out in patients' lives will help physicians fulfil their role as prescribers.[3,19] This study aimed to explore how patients with experience of acute coronary heart disease make sense of, and deal with, the fact of being prescribed cardiovascular preventive medication.

Methods

Setting and participants

Participants were recruited consecutively from COR-PRIM, a primary care study of self-care after acute coronary heart disease.[26] In this population, there are experiences of previous prescriptions of CPM both for primary and secondary prevention, making it suitable for our study aim. Out of 29 approached by telephone, 22 accepted to be interviewed. One interview was excluded because the audio-recording failed. See Table 1 for participant characteristics.

Table 1. Participant characteristics, N = 21

**Characteristics**	**n**
**Sex**	
Male	17
Female	4
**Age, years**	72 (52-86)[1]
**Residential area**	
City	8
Rural or small town	13
**Education**	
Compulsory education[2]	8
Upper secondary school	6
University	7
**Marital status**	
Cohabitating	16
Living alone	5

[1] Median (min-max)
[2] Less than 10 years in school

The sample size was preliminary set a priori to around twenty, based on the method used.[27] In analysing, we found the data set do be rich and varied, and decided not to include more participants. All participants had been admitted to hospital for acute coronary heart disease 6-12 months prior to the interview, and gave written consent to

For peer review only

participate after receiving oral and written information about the study. The wives of six of the participants were present during the interviews, and supported their husbands to recall events and express their experiences. The participants were interviewed in their homes by JH. She is a general practitioner with no prior relation to any of the participants. They were asked to recall a situation in which cardiovascular preventive drugs had been discussed or prescribed, without distinguishing between primary and secondary prevention. Drugs considered were antihypertensive medication, statins, and antiplatelet drugs such as acetylsalicylic acid. Follow-up questions were asked to guide towards relevant topics and to explore them in greater depth. Figure 1 presents the interview guide. The interviews lasted for 45-90 minutes, and were audio-recorded and transcribed verbatim by JH.

Analysis

We chose a narrative approach to analyse patients' accounts of being prescribed CPM.[27,28] The analysis concerned both content (*what* was told), and structure (*how* it was told) as described by Reissman.[27] In the initial reading of the transcriptions, we looked for salient features of the material as a whole, with a narrative framework in mind. Structural elements (Figure 2) served to identify narratives.[28] The prescription of CPM was the complicating action around which these narratives revolved. The narratives described both the prescription of CPM after a recent cardiovascular event, and previous occasions on which CPM had been prescribed. Some interviews contained more than one narrative. A synopsis of each interview was made, in which the narratives were reproduced in condensed form. These condensed narratives were analysed in greater depth to define experiences, deliberations, and actions of the participants related to the fact that they were being prescribed CPM. Structural features such as the framings and evaluations of the narrative material displayed their sense-making of reported speech.[28]

The narratives were rich in quotes. The participants told about what had been said, but also what may be said in a future scenario, by characters such as prescribers, other physicians, nurses, relatives and friends. They quoted texts, such as information leaflets. They also quoted themselves, both actual utterances, and their thoughts: their inner voices. Opinions and experiences of phenomena in the narratives became evident through internal evaluations.[28] Some, for example, used a mocking tone of voice when

For peer review only

quoting someone they did not agree with. The analysis of the different voices used by the participants rested on Goffman's analytical framework for aspects of narrators, including the concept of *"footing".* This framework helps to reveal the speakers' positioning of themselves in relation to reported speech and events.[29]

Tentative themes within one interview were assessed against each other, and then against those from other interviews. Similar tentative themes were brought together into more general themes, which were then further elaborated in an iterative process. Finally, the themes were checked for their bearing on the original interview data, and adjusted to provide the resulting themes. In each step, JH made an initial analysis. All authors discussed the emerging results. Throughout the process, the analytical focus was to capture the participants' ways of dealing with the fact that they were being prescribed CPM.

Patient and public involvement

No patient involvement.

Results

The interviews were numbered #1-21, in the order they were conducted. The participants' medical histories ranged from no CPM prescriptions prior to the recent coronary event (#1), to over twenty years since the first myocardial infarction (#7), and many years with primary preventive medication with (#15) or without type two diabetes (#11). Four themes became apparent during the analysis: *"a matter of living"*, *"reconciliation of conflicting self images", "being in the hands of expertise",* and *"taking medicines no longer a big deal".* Quotes, including longer passages, were selected to illustrate the themes, and to display the dialogic and narrative structure of the data.

A matter of living

Implicit in the recommendation for preventive treatment is its importance for survival. Such a message may evoke an awareness of being mortal, and the fundamental will to live. Dealing with the awareness of mortality is not merely about avoiding death, but involves reflections about what makes life worth living, and what it means to lead a meaningful life. Staying alive to continue relationships with loved ones was salient:

For peer review only - http://bmjopen.bmj.com/site/about/guidelines.xhtml

I didn't use to take that much medication but now I have to.
Interviewer: Who makes you?
The COPD (chronic obstructive pulmonary disease) and heart
Wife: Basically, he wants to live ...
... with you! (looks at wife) (#12)

The awareness of being mortal that was evoked by being prescribed CPM stimulated efforts to strike a balance between the pros and cons of taking medication. For example, side effects could be accepted with the argument that the medication was regarded lifesaving:

It is important that I take this and I don't care if it makes me feel a bit sick, I have to or else I will die. (#14)

Some participants held fundamental objections to taking CPM, but even so experienced gratitude for having the chance to stay alive, which was attributed to the medication:

... Now you're a bit scared and think: well, I'd better take it for the heart, that's what they said ... so of course you're glad for all these pills and that they work in the body. (#2)

Reconciliation of conflicting self-images

Most participants were generally averse to taking medicines, and held the view that they are generally overprescribed and overused. The participants typically presented themselves as sceptical towards medication, not prone to using it, and stated that medicines should only be taken if necessary. Being given a prescription and becoming a "pill-taker" challenged this self-image. Most participants had been prescribed CPM for primary or secondary prevention, prior to the recent hospitalisation for acute coronary heart disease. Both participants who had been prescribed medication for primary prevention before having had symptomatic cardiovascular disease, and those who didn't have it until after such disease, became aware that they were at risk of future cardiovascular disease. They perceived that their status changed from healthy to being in need of correction through medical treatment:

(Now) I have to. I take it for self-preservation, but like I say: I don't like to put chemicals into my body. (#3)

For peer review only - http://bmjopen.bmj.com/site/about/guidelines.xhtml

One way of dealing with the new conditions was to seek to preserve the "only-when-necessary" view of self. Taking CPM was framed as an inevitable consequence of the state of health, not as an active choice. A face-saving rhetoric indicated that this was a sensitive topic, which evoked feelings of embarrassment and shame. Potential blame for having caused the state of being at risk was countered by a defensive line of argument. Participants emphasised that they had been struck by disease, or had high cholesterol values, in spite of a healthy lifestyle. Having smoked was mentioned, but downplayed: *"I used to smoke but I quit … I used to drink a lot of milk … I think that was the biggest cause" (#21).* Hard work, and stressful life events were common explanations. Heredity was another: *"These bloody sickness-genes" (#11).* One line of defence was to claim to be an exception in comparisons with others: *"Many get way too much medicine, but that's perhaps more in nursing homes" (#4),* while another was to normalise the condition: *"Now it (angioplasty) is common, they go: 'Oh you did it too?' … In the nineties you went down in the basement of the University Hospital. It was like coming to Hades in the underworld and was serious as hell. They did a couple a week, now they do several a day." (#7)*

Being in the hands of expertise

A personal relationship with a prescriber was essential for patients in their work to seek reassurance that CPM was necessary. Some had experienced a continuous relationship with the physician responsible for prescribing their medication, but this was not the case for most. Without such a relationship, patients experienced feelings of abandonment:

"They took away furosemide and that other one, and I lacked … they just said: 'You can see how you feel', but how and when, with whom should I talk about how I feel? I didn't have anyone to talk with; it was all up to me." (#15)

Being in the hand of expertise and *feel* powerless may not equate to *being* powerless, when patients take a position in which they actively accept being cared for. Patients may choose to place the responsibility for decision-making on the prescribing doctor if they have a trustful relationship with the doctor, and in this way resolve their doubts about whether it is necessary to take medication:

For peer review only - http://bmjopen.bmj.com/site/about/guidelines.xhtml

"You look up to someone who knows everything about this, and you are grateful that someone sort of weighs and measures you, and corrects and changes the medicines. It's like going to the dentist. You're helpless. What if the dentist says: 'Hey this tooth has to come out'. You don't ask: 'Do I really have to?'. Or at the garage, you don't have a clue; the car mechanic can say anything. So it feels a bit like you are powerless." (#1)

The question *"Do I really have to take it?"* occurred frequently in the narratives. Reassurance was sought in dialogue with a prescribing physician, who could explain sufficiently clearly why the medication was recommended, and who was willing to take the responsibility to state that it was necessary. The attitude towards the authority of expertise ranged from more-or-less considered acceptance and trust: "*You have to trust what they say, and they know that a lot better than I do anyway." (#4)*, to critical scepticism: "*It is in my nature. I don't just accept anything" (#16).* Many narratives illustrated how important it was to have an expert to turn to, regardless of how the patient viewed the authority of expertise. This was particularly the case when such an expert was lacking. Some patients were aware of treatment guidelines, and that the expert community has different views on treatment indications. In spite of this, and in spite of an expressed request for information, patients described themselves to be in the hands of the medical expertise. Most were content with simply the possibility of dialogue: "*You don't pick a fight about it and say: 'I won't take this', or: 'I want this that I read about'. No, you take it for granted that this is a competent person." (#19)*

Taking medicines no longer a big deal

The concluding remarks in many narratives showed that an attitude in which taking medicines was no longer a big deal had been acquired. Medication had been incorporated into ordinary life:

They said you had to take this ... for everything to work and for the blood clots that can build up and all that, and I have brochures about it that I can read if I want, but I think now when you have them sitting there (nods towards the medicine packages on the kitchen shelf) it goes automatically, every night and every morning. (#2)

For peer review only - http://bmjopen.bmj.com/site/about/guidelines.xhtml

For some, a no-big-deal attitude came with minimal effort or reflection, whereas most had put considerable work into dealing with being prescribed CPM. This process of becoming a "pill-taker" without it being a big deal seemed to be facilitated by the resolution of existential challenges, the reconciliation of conflicting self-images, and the conclusion, after dialogue with a trusted prescriber, that it was necessary. The recent coronary heart event further lessened any hesitation about taking CPM, and motivated the view that it was truly necessary:

Nobody had a clue before this happened or talked about me belonging to a risk group, but now after getting the stent, I obviously do, so that is something to think about (when deciding about cholesterol treatment). (#19)

Some patients who were still in the process of dealing with one or several aspects of being prescribed CPM experienced their medication as a reminder of their vulnerability. It was difficult to ignore these feelings. Taking the medicines was still burdensome, and not yet an effortless daily routine:

(Shows that she has put the medicine dispenser out of sight in a cupboard) Sometimes when it is time for medication, and there are people over, I think I mustn't forget to take it when they have left, because I would never … Yak, it's so dreadful … Perhaps it takes a year to grasp, before everything has calmed down, all of this, and you make it sort of into a ritual after a while. (#1)

Discussion

Dealing with being prescribed CPM involved the themes: "*A matter of living*, "*Reconciliation of conflicting self-images*", "*Being in the hands of expertise*", and "*Taking medicines no longer a big deal*". Patients oriented towards living rather than survival, and to find a coherent view of self as someone who must take CPM. The decision about CPM was framed as a question about whether it was necessary, not as a patient choice. Patients sought an answer from a reliable prescriber to the implicit question: Is this medication really necessary for me? This implies that patients may execute their autonomy by actively placing the responsibility for the decision about CPM with the prescriber. A successful process resolved doubts about if taking CPM was necessary and

For peer review only - http://bmjopen.bmj.com/site/about/guidelines.xhtml

seemed to facilitate the incorporation of it into everyday life. A stalled process of making it necessary appeared as a particular burden of treatment.

The wide range of medical histories represented is a strength of this study. Previous prescriptions, both for primary and secondary prevention, were contrasted to the most recent, and reflected in the light of the recent hospitalisation, which contributes to the credibility of the results. The participants probably still experienced reactions to the serious event, which may have resulted in their focus on existential matters.[30] Apart from the connection between the recent hospitalization and secondary preventive medication the patient experiences from primary and secondary prevention were similar. This is in line with previous studies of patient perspectives on CPM.[5,6,9] The COR-PRIM participants rated high on scales that measure patient empowerment and general self-efficacy.[31] They may be inclined to share their viewpoints to improve healthcare, and particularly well-suited to give voice to general experiences of receiving prescriptions for CPM, although possibly not representative of less empowered patients. The interviews were rich in content, with reflections on experiences of being prescribed CPM that may be transferable to similar contexts. Non-adherence was disclosed, and negative experiences of encounters with doctors reported, indicating that the participants felt trust, and did not adjust their stories to please the interviewer. All themes appeared in almost every interview, suggesting that the themes have a general relevance. The presence of partners in six interviews was not planned, but turned out to be advantageous. As noted in a similar study, the dialogues of the couples added perspectives to the accounts, while keeping a respectful focus on the patient´s experiences.[9] Some participants showed signs of cerebrovascular disease with slight cognitive impact. This is to be expected in this group of patients. It is important, and a strength of the study that also their voices are heard. The presence of partners facilitated this. We believe that one reason to participate in COR-PRIM was the lack of a personal relationship with a prescribing physician that many participants described. Health care in Sweden has a lower proportion of general practitioners than similar countries, and only a minority of the population are registered with a regular GP.[32] This may reduce the transferability of the findings. On the other hand, the negative experiences of lacking a continuous personal relationship with a prescriber that the participants voiced suggest that this is of general importance.

For peer review only - http://bmjopen.bmj.com/site/about/guidelines.xhtml

The general reluctance towards medication was overridden by the will to live, with an understanding of taking CPM as necessary to survive. It was framed as an inevitable must, not as a choice. Polak *et al.* described this standpoint as a defence of morality, i.e. against the accusations of leading an unhealthy life style.[9] This agrees with the defensive rhetoric that many of the participants took, and the way in which they dealt with the need for CPM as a morally sensitive issue. The framing of it being necessary to take CPM appeared also to overrule uncertainty, making it into a question of life or death, not quite consistent with the notion of choice between alternatives in commonly advocated shared decision-making models. The effects of CPM are overestimated by both laymen and general practitioners, which may be a result of this concept of necessity.[6,16] Patients must deal with a challenge to the self-image, and self-perception changes when long-term medication such as CPM is prescribed.[11] Some of the resistance to taking medication may stem from a denial of being ill.[1] However, also medication prescribed for prevention, in the absence of symptomatic disease, may call for a rewriting of the biography.[12] The key to the reconciliation of the conflicting images held by the patients in our study was the work with identity by which they established that it was necessary to take CPM, and that they had become a medicine-taker.

Our findings indicate that there are patients who want explanations about CPM, but want the prescriber to determine whether it is necessary. It agrees with previous work where patients prefer to take part in the decision-making process, but not to make the final decision.[33,34] Patients can execute their agency and autonomy by asking for information and a dialogue but deferring the responsibility for the decision.[13] Prescribers must be aware of the inherent asymmetry in a patient-doctor relationship, and be receptive to the patients' active deferral of responsibility for decision-making. They must strike a balance: to avoid violating patient autonomy without abandoning them.[35,36] Patient autonomy in this situation is not absolute, but depends on the response of the prescriber.[20,37] Elderly patients who take several medicines are more likely to find the medication necessary if they interact with a "good" doctor.[33] Even healthcare professionals, when patients, depend on the relationship with doctors and nurses, find rest in their hands, and trust that they know what they're doing.[38] Plain

For peer review only - http://bmjopen.bmj.com/site/about/guidelines.xhtml

information from a faceless expert community causes even better-informed patients to feel abandoned.[39] The process of making the taking of CPM to be necessary may result in the taking being incorporated into ordinary everyday life, even though it may threaten the "ordinariness" for persons who view themselves as non-pill-takers, and those who view medicines as a necessary evil.[8,9]

The implicit or explicit question: "Is it really necessary for me to take CPM?" requires a definitive answer. The submission to expertise may be a conscious and autonomous act by which responsibility for decisions is placed on the professional. A personal continuous relationship with the prescriber helps patients to express their preferences and needs during the process that leads to a decision, and eventually to a no-big-deal attitude towards taking CPM. As Julian Tudor Hart put it: *"Effective health care has to be built around real continuing personal stories, not episodic fragments of standardized process."*.[40] In the same spirit, the concept of shared understanding has been proposed, in an attempt to merge SDM, evidence-based medicine, and patient-centred medicine. Attentive listening to patients' narratives is essential to the practice of shared understanding.[25,41]

Conclusions

Based on the themes "*A matter of living"* and "*Reconciliation of conflicting self-images",* we suggest that unmet existential needs when being prescribed CPM, is a component of the burden of treatment. Inferred from the themes "*Being in the hands of expertise* and *Taking medicines no longer a big deal",* we propose that a continuous and trustful relationship with the prescribing doctor may facilitate the reconciliation of conflicting self-images, and support patients in their efforts to incorporate their medicines taking into daily life. These findings should be investigated in larger populations for increased knowledge of how common the different ways of dealing with being prescribed CPM are in different groups of patients.

Competing interests

The authors declare no conflicts of interests.

For peer review only - http://bmjopen.bmj.com/site/about/guidelines.xhtml

Ethical approval

Ethical approval was obtained from the Regional Ethics Committee of Linköping, Ref. No. 2014/5-31.

Author contributions

CER, JH, AKK and SN were responsible for the planning and design of the study. AKK and SN were responsible for the recruitment of participants. JH performed and transcribed the interviews. JH, CER, AKK and SN contributed to the analysis. JH wrote a manuscript draft, which CER, AKK and SN critically revised. All authors approve of the submission of this manuscript and agree to be accountable for all aspects of the work in ensuring that questions related to the accuracy or integrity of any part of the work are appropriately investigated and resolved.

Funding

This study was supported by the County Council of Östergötland (no grant number) and Lions forskningsfond mot folksjukdomar (Lions Sweden, Districts 101-V, 101-S, 101-O and Linköping University Medical Faculty, no grant number).

Data availability statement

No additional data available.

Acknowledgments

We are indebted to the participants for their generosity with their time and stories, and for their hospitality during the interviews.

Tables and figures

Table 1 Participant characteristics.

Figure 1 Interview guide.

Figure 2 Structural elements in narratives according to Labov & Waletsky.

REFERENCES

1 Pound P, Britten N, Morgan M, *et al.* Resisting medicines: a synthesis of qualitative studies of medicine taking. *Soc Sci Med 1982* 2005;**61**:133–55. doi:10.1016/j.socscimed.2004.11.063

For peer review only

2 Horne R, Weinman J, Hankins M. The beliefs about medicines questionnaire: The development and evaluation of a new method for assessing the cognitive representation of medication. *Psychol Health* 1999;**14**:1–24.

3 May CR, Eton DT, Boehmer K, *et al.* Rethinking the patient: using Burden of Treatment Theory to understand the changing dynamics of illness. *BMC Health Serv Res* 2014;**14**:281. doi:10.1186/1472-6963-14-281

4 Leppin AL, Montori VM, Gionfriddo MR. Minimally Disruptive Medicine: A Pragmatically Comprehensive Model for Delivering Care to Patients with Multiple Chronic Conditions. *Healthcare* 2015;**3**:50–63. doi:10.3390/healthcare3010050

5 Jansen J, McKinn S, Bonner C, *et al.* Shared decision-making about cardiovascular disease medication in older people: a qualitative study of patient experiences in general practice. *BMJ Open* 2019;**9**:e026342. doi:10.1136/bmjopen-2018-026342

6 Lytsy P, Burell G, Westerling R. Views on treatment necessity, harm, and benefits in patients using statins. *Med Decis Mak Int J Soc Med Decis Mak* 2010;**30**:594–609. doi:10.1177/0272989X09353196

7 Gillett J, Ross A. Confronting Medicine's Dichotomies: Older Adults' Use of Interpretative Repertoires in Negotiating the Paradoxes of Polypharmacy and Deprescribing. *Qual Health Res* 2019;:1049732319868981. doi:10.1177/1049732319868981

8 Eborall HC, Will CM. 'Prevention is better than cure, but ...': Preventive medication as a risk to ordinariness? *Health Risk Soc* 2011;**13**:653–68. doi:10.1080/13698575.2011.624177

9 Polak L. What is wrong with 'being a pill-taker'? The special case of statins. *Sociol Health Illn* 2017;**39**:599–613. doi:10.1111/1467-9566.12509

10 Köhler AK, Nilsson S, Jaarsma T, *et al.* Health beliefs about lifestyle habits differ between patients and spouses 1 year after a cardiac event - a qualitative analysis based on the Health Belief Model. *Scand J Caring Sci* 2017;**31**:332–41. doi:10.1111/scs.12351

11 Andén A, Andersson S-O, Rudebeck C-E. Satisfaction is not all – patients' perceptions of outcome of general practice consultations, a qualitative study. *BMC Fam Pract* 2005;**6**:43. doi:10.1186/1471-2296-6-43

12 Sångren H, Reventlow S, Hetlevik I. Role of biographical experience and bodily sensations in patients' adaptation to hypertension. *Patient Educ Couns* 2009;**74**:236–43. doi:10.1016/j.pec.2008.08.007

13 Hultberg J, Rudebeck CE. Patient participation in decision-making about cardiovascular preventive drugs – resistance as agency. *Scand J Prim Health Care* Published Online First: 28 February 2017.http://www.tandfonline.com/doi/full/10.1080/02813432.2017.1288814 (accessed 1 Mar 2017).

For peer review only

14 Landmark AMD, Gulbrandsen P, Svennevig J. Whose decision? Negotiating epistemic and deontic rights in medical treatment decisions. *J Pragmat* 2015;**78**:54–69. doi:10.1016/j.pragma.2014.11.007

15 Hultberg J, Rudebeck CE. Clinical Gaze in Risk-Factor Haze: Swedish GPs' Perceptions of Prescribing Cardiovascular Preventive Drugs. *Int J Fam Med*;**2012**:612572. doi:10.1155/2012/612572

16 Treadwell JS, Wong G, Milburn-Curtis C, *et al.* GPs' understanding of the benefits and harms of treatments for long-term conditions: an online survey. *BJGP Open* Published Online First: 4 March 2020. doi:10.3399/bjgpopen20X101016

17 Bomhof-Roordink H, Gärtner FR, Stiggelbout AM, *et al.* Key components of shared decision making models: a systematic review. *BMJ Open* 2019;**9**:e031763. doi:10.1136/bmjopen-2019-031763

18 Ofstad EH, Frich JC, Schei E, *et al.* Temporal characteristics of decisions in hospital encounters: A threshold for shared decision making? A qualitative study. *Patient Educ Couns* 2014;**97**:216–22. doi:10.1016/j.pec.2014.08.005

19 Weiner SJ, Schwartz A. Contextual Errors in Medical Decision Making: Overlooked and Understudied. *Acad Med J Assoc Am Med Coll* 2016;**91**:657–62. doi:10.1097/ACM.0000000000001017

20 Herlitz A, Munthe C, Törner M, *et al.* The Counseling, Self-Care, Adherence Approach to Person-Centered Care and Shared Decision Making: Moral Psychology, Executive Autonomy, and Ethics in Multi-Dimensional Care Decisions. *Health Commun* 2016;**31**:964–73. doi:10.1080/10410236.2015.1025332

21 Beauchamp TL, Childress JF. *Principles of biomedical ethics*. Oxford: : Oxford University Press 2001.

22 Piepoli MF, Hoes AW, Agewall S, *et al.* 2016 European Guidelines on cardiovascular disease prevention in clinical practice. *Atherosclerosis* 2016;**252**:207–74. doi:10.1016/j.atherosclerosis.2016.05.037

23 Légaré F, Adekpedjou R, Stacey D, *et al.* Interventions for increasing the use of shared decision making by healthcare professionals. *Cochrane Database Syst Rev* 2018;**7**:CD006732. doi:10.1002/14651858.CD006732.pub4

24 Merchant FM, Dickert NW, Howard DH. Mandatory Shared Decision Making by the Centers for Medicare & Medicaid Services for Cardiovascular Procedures and Other Tests. *JAMA* 2018;**320**:641–2. doi:10.1001/jama.2018.6617

25 Locock L, Lehman R, Epstein RM. Sharing Experiences of Illness and Care. *JAMA Intern Med* 2017;**177**:1249–50. doi:10.1001/jamainternmed.2017.1935

26 Karner A, Nilsson S, Jaarsma T, *et al.* The effect of problem-based learning in patient education after an event of CORONARY heart disease--a randomised study in PRIMARY health care: design and methodology of the COR-PRIM study. *BMC Fam Pr*;**13**:110. doi:10.1186/1471-2296-13-110

For peer review only

For peer review only - http://bmjopen.bmj.com/site/about/guidelines.xhtml

27 Riessman CK. *Narrative methods for the human sciences*. Los Angeles, Calif.: : Sage 2008.

28 Labov W, Waletzky J. Narrative analysis: Oral versions of personal experience. *J Narrat Life Hist* 1997;**7**:3–38. doi:10.1075/jnlh.7.02nar

29 Goffman E. *Forms of talk*. Philadelphia: : Univ. of Pennsylvania Press 2008.

30 Odell A, Grip L, Hallberg LR-M. Restenosis after Percutaneous Coronary Intervention (PCI): Experiences from the Patients' Perspective. *Eur J Cardiovasc Nurs* 2006;**5**:150–7. doi:10.1016/j.ejcnurse.2005.10.004

31 Kärner Köhler A, Tingström P, Jaarsma T, *et al.* Patient empowerment and general self-efficacy in patients with coronary heart disease: a cross-sectional study. *BMC Fam Pract* 2018;**19**:76. doi:10.1186/s12875-018-0749-y

32 International Health Policy Survey of Primary Care Physicians 2015. https://www.vardanalys.se/in-english/reports/international-health-policy-survey-of-primary-care-physicians-2015 (accessed 8 Aug 2020).

33 Moen J, Bohm A, Tillenius T, *et al.* "I don't know how many of these [medicines] are necessary.."—A focus group study among elderly users of multiple medicines. *Patient Educ Couns* 2009;**74**:135–41. doi:10.1016/j.pec.2008.08.019

34 Edwards A, Elwyn G. Inside the black box of shared decision making: distinguishing between the process of involvement and who makes the decision. *Health Expect* 2006;**9**:307–20. doi:10.1111/j.1369-7625.2006.00401.x

35 Morberg Jämterud S. Human Dignity : A Study in Medical Ethics. Published Online First: 2016.http://urn.kb.se/resolve?urn=urn:nbn:se:uu:diva-300409 (accessed 26 Nov 2018).

36 Lindström A, Weatherall A. Orientations to epistemics and deontics in treatment discussions. *J Pragmat* 2015;**78**:39–53. doi:10.1016/j.pragma.2015.01.005

37 Gulbrandsen P, Clayman ML, Beach MC, *et al.* Shared decision-making as an existential journey: Aiming for restored autonomous capacity. *Patient Educ Couns* Published Online First: 6 July 2016. doi:10.1016/j.pec.2016.07.014

38 Olthuis G, Leget C, Grypdonck M. Why shared decision making is not good enough: lessons from patients. *J Med Ethics* 2014;**40**:493–5. doi:10.1136/medethics-2012-101215

39 Johansson M, Jørgensen KJ, Getz L, *et al.* "Informed choice" in a time of too much medicine—no panacea for ethical difficulties. *BMJ* 2016;**353**:i2230. doi:10.1136/bmj.i2230

40 Hart JT. *The political economy of healthcare: where the NHS came from and where it could lead*. Bristol: : Policy Press 2010.

For peer review only

41 Maskrey N, Gordon A. Shared Understanding With Patients. *JAMA Intern Med* 2017;**177**:1247–8. doi:10.1001/jamainternmed.2017.1932

For peer review only

Figure 1. Interview guide

Topics to cover in interviews:

- Cardiovascular risk, risk factors, the concept of risk and the participant's own risk of cardiovascular disease
- Power, responsibility and participation in prescribing decisions
- Knowledge relevant for treatment decisions
- Communication between patients and healthcare professionals about medication

For peer review only

Figure 2. Structural elements in narratives according to Labov & Waletsky

**Abstract**	introduction of story
**Orientation**	background information, setting and participants in story
**Complicating action**	account for events in temporally ordered narrative clauses
**Evaluation**	revelations of attitudes towards the content of the story, what the point of the story is according to the narrator, why it makes sense, how it creates meaning
**Resolution**	the end result of the events
**Coda**	concluding remark

For peer review only

COREQ checklist with comments and references to manuscript.

Manuscript: bmjopen-2021-056677

Title: Being prescribed cardiovascular preventive medication – a narrative analysis of interviews with patients with recent acute coronary heart disease.

Checklist:

Consolidated criteria for reporting qualitative studies (COREQ)

From: Allison Tong, Peter Sainsbury, Jonathan Craig, Consolidated criteria for reporting qualitative research (COREQ): a 32-item checklist for interviews and focus groups, *International Journal for Quality in Health Care*, Volume 19, Issue 6, December 2007, Pages 349–357, https://doi.org/10.1093/intqhc/mzm042

No. Item	Guide questions/description	Page, line #
**Domain 1: Research team and reflexivity**		
Personal Characteristics		
1. Interviewer/facilitator	Which author/s conducted the interview or focus group?	P6, line 4
2. Credentials	What were the researcher's credentials? *E.g. PhD, MD*	P6, line 4
3. Occupation	What was their occupation at the time of the study?	P6, line 4
4. Gender	Was the researcher male or female?	P6, line 4
5. Experience and training	What experience or training did the researcher have?	P6, line 4
Relationship with participants		
6. Relationship established	Was a relationship established prior to study commencement?	P6, line 4
7. Participant knowledge of the interviewer	What did the participants know about the researcher? *e.g. personal goals, reasons for doing the research*	P6, line 4
8. Interviewer characteristics	What characteristics were reported about the interviewer/facilitator? *e.g. Bias, assumptions, reasons and interests in the research topic*	P6, line 4<br>P12, lines 16-18

For peer review only

**Domain 2: study design**		
Theoretical framework		
9. Methodological orientation and Theory	What methodological orientation was stated to underpin the study? *e.g. grounded theory, discourse analysis, ethnography, phenomenology, content analysis*	P6, line 6 and onwards
Participant selection		
10. Sampling	How were participants selected? *e.g. purposive, convenience, consecutive, snowball*	P5, line 10 and onwards
11. Method of approach	How were participants approached? *e.g. face-to-face, telephone, mail, email*	P5 line 13
12. Sample size	How many participants were in the study?	P5 line 14 and table 1
13. Non-participation	How many people refused to participate or dropped out? Reasons?	P5 line 13-14
Setting		
14. Setting of data collection	Where was the data collected? *e.g. home, clinic, workplace*	P6, line 4
15. Presence of non-participants	Was anyone else present besides the participants and researchers?	P6, line 1
16. Description of sample	What are the important characteristics of the sample? *e.g. demographic data, date*	P5. Table 1
Data collection		
17. Interview guide	Were questions, prompts, guides provided by the authors? Was it pilot tested?	P6, line 5 and Figure 1
18. Repeat interviews	Were repeat interviews carried out? If yes, how many?	No.
19. Audio/visual recording	Did the research use audio or visual recording to collect the data?	P6, line 10
20. Field notes	Were field notes made during and/or after the interview or focus group?	No
21. Duration	What was the duration of the interviews or focus group?	P6 line 10
22. Data saturation	Was data saturation discussed?	P5, line 23
23. Transcripts returned	Were transcripts returned to participants	No

For peer review only

	for comment and/or correction?	
**Domain 3: analysis and findings**		
Data analysis		
24. Number of data coders	How many data coders coded the data?	P7, line 9
25. Description of the coding tree	Did authors provide a description of the coding tree?	No
26. Derivation of themes	Were themes identified in advance or derived from the data?	P7, line 5
27. Software	What software, if applicable, was used to manage the data?	Not applicable
28. Participant checking	Did participants provide feedback on the findings?	No
Reporting		
29. Quotations presented	Were participant quotations presented to illustrate the themes/findings? Was each quotation identified? *e.g. participant number*	P7, line 17 and onwards (results section)
30. Data and findings consistent	Was there consistency between the data presented and the findings?	P7, line 17 and onwards (results section)
31. Clarity of major themes	Were major themes clearly presented in the findings?	P7, line 17 and onwards (results section)
32. Clarity of minor themes	Is there a description of diverse cases or discussion of minor themes?	No, not applicable.

For peer review only
